# Supplementary figures and images for: Toward streamline variant classification: discrepancies in variant nomenclature and syntax for ClinVar pathogenic variants across annotation tools
Source: Hum Genomics. 2025 Jun 21;19:70. doi: 10.1186/s40246-025-00778-x (PMC12181866; doi:10.1186/s40246-025-00778-x)

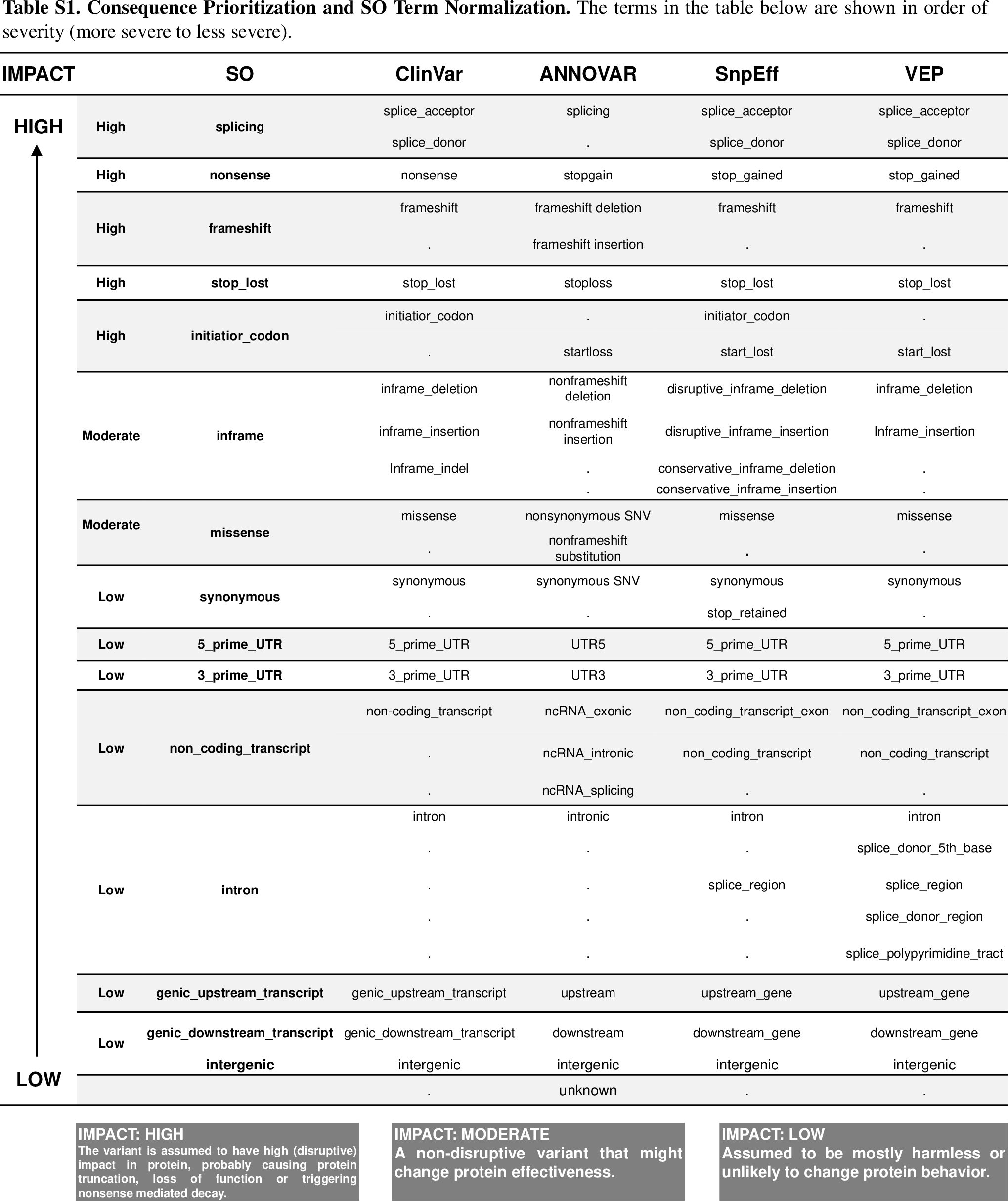

Supplement: Supplementary file 1 — Additional file 1 [file 40246_2025_778_MOESM1_ESM.tif]

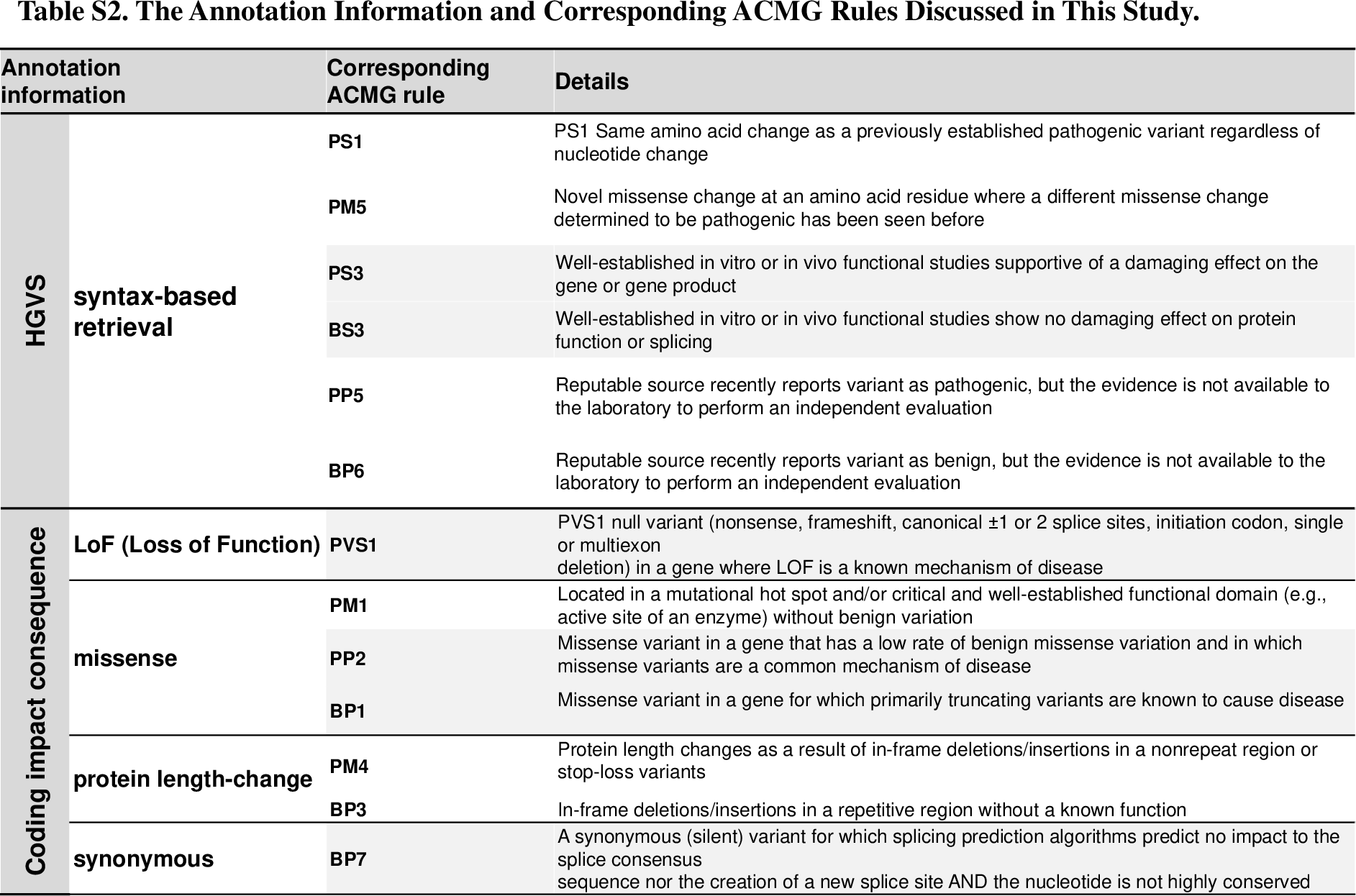

Supplement: Supplementary file 2 — Additional file 2 [file 40246_2025_778_MOESM2_ESM.tif]

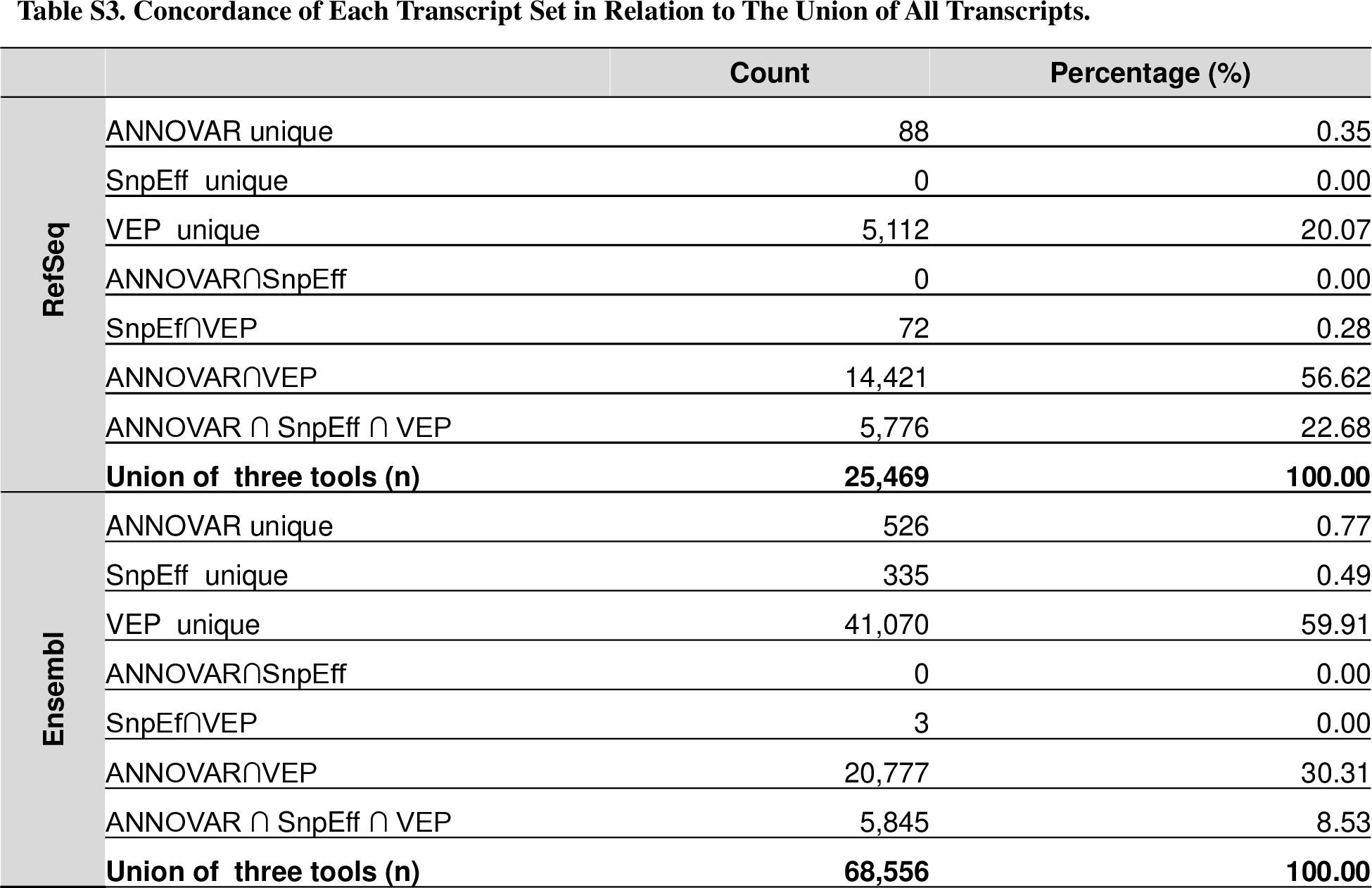

Supplement: Supplementary file 3 — Additional file 3 [file 40246_2025_778_MOESM3_ESM.tif]

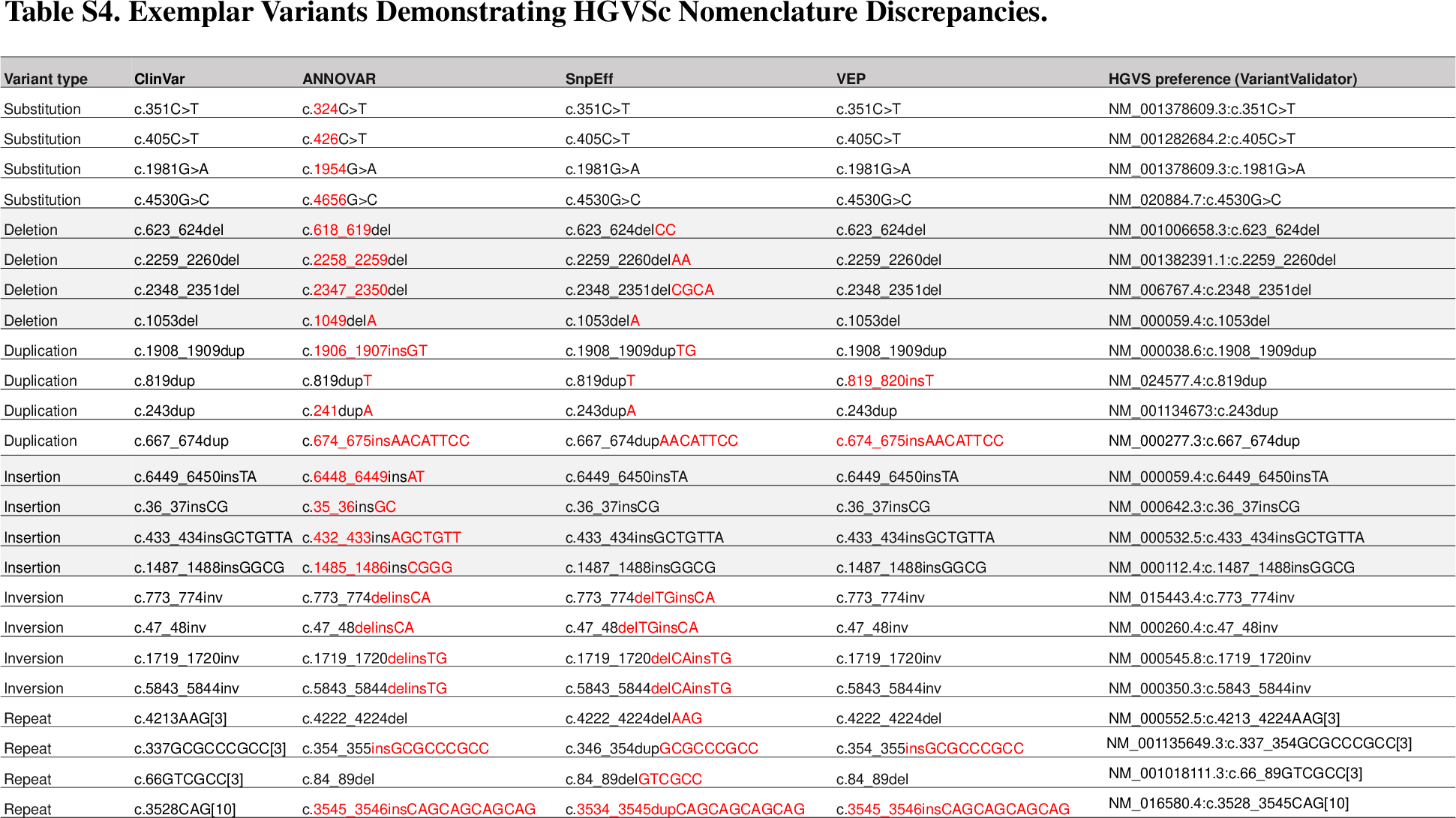

Supplement: Supplementary file 4 — Additional file 4 [file 40246_2025_778_MOESM4_ESM.tif]

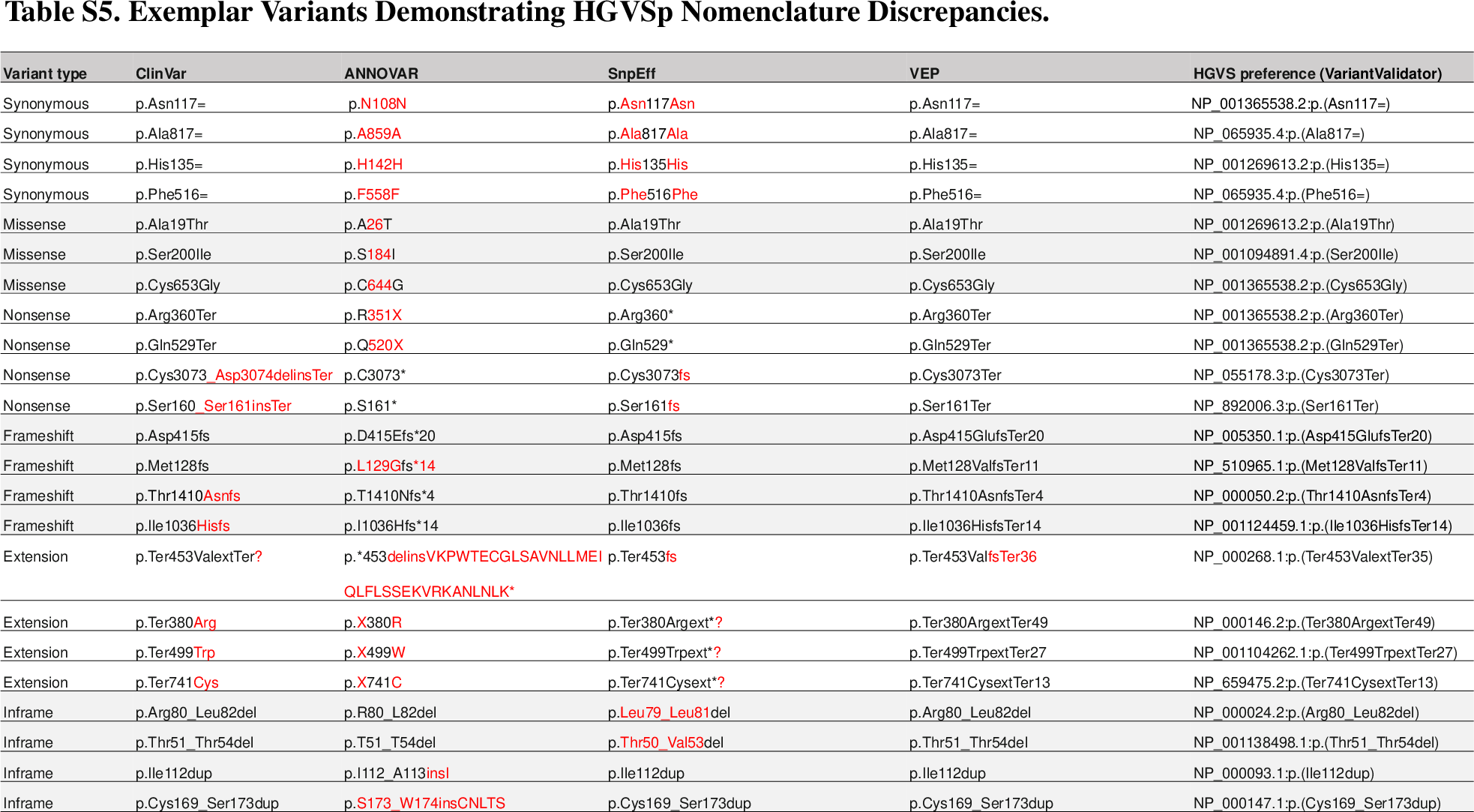

Supplement: Supplementary file 5 — Additional file 5 [file 40246_2025_778_MOESM5_ESM.tif]

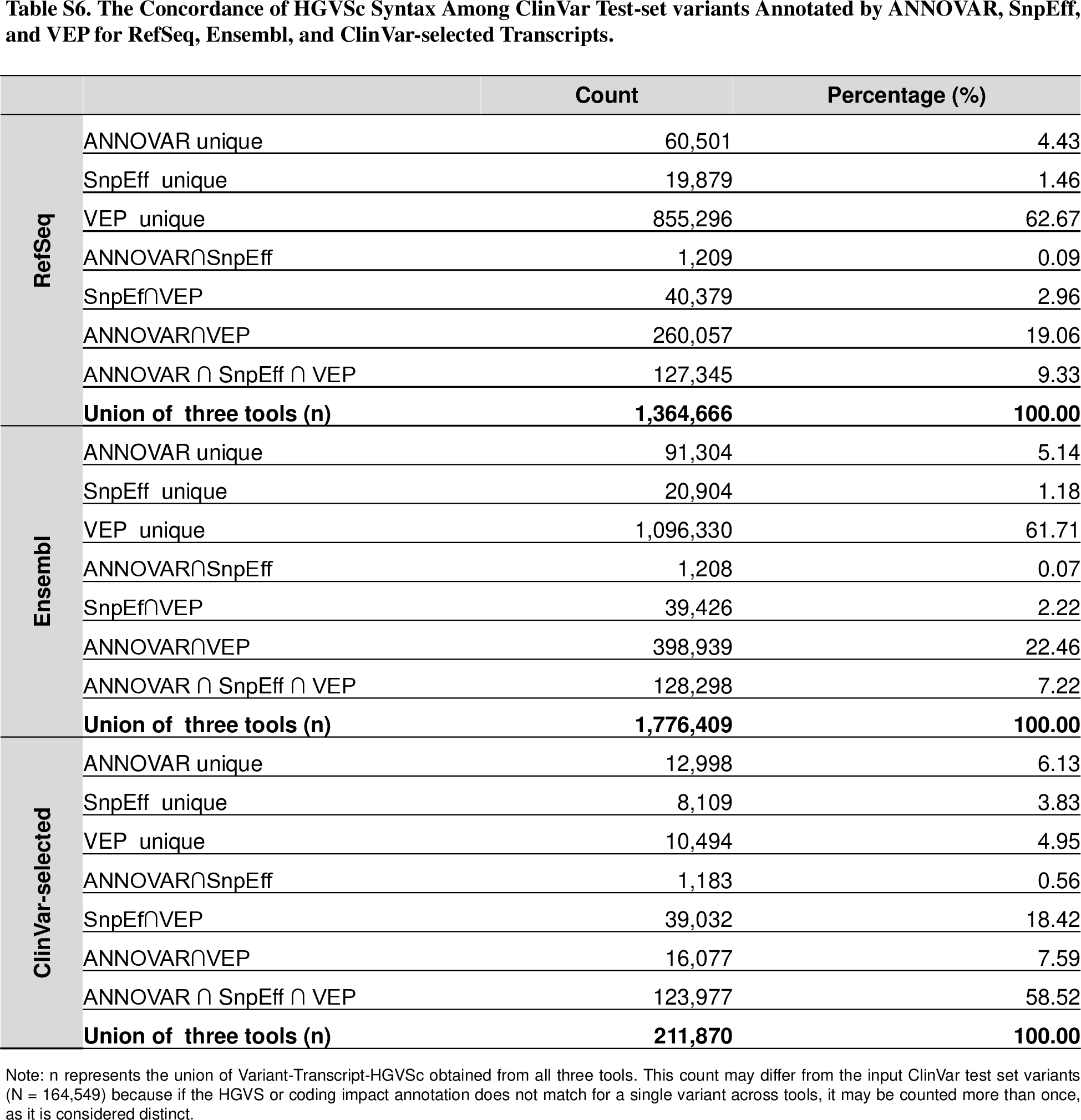

Supplement: Supplementary file 6 — Additional file 6 [file 40246_2025_778_MOESM6_ESM.tif]

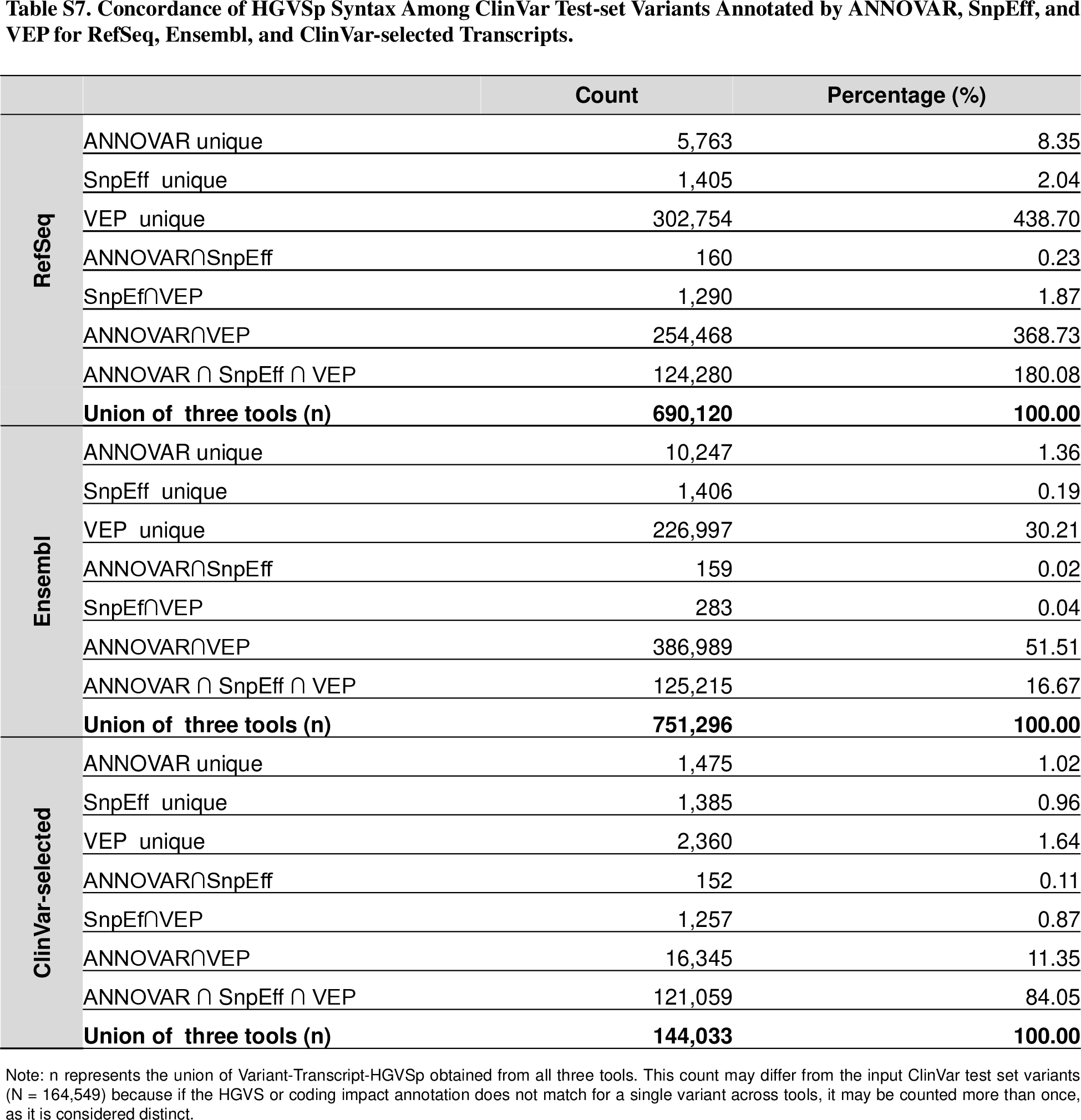

Supplement: Supplementary file 7 — Additional file 7 [file 40246_2025_778_MOESM7_ESM.tif]

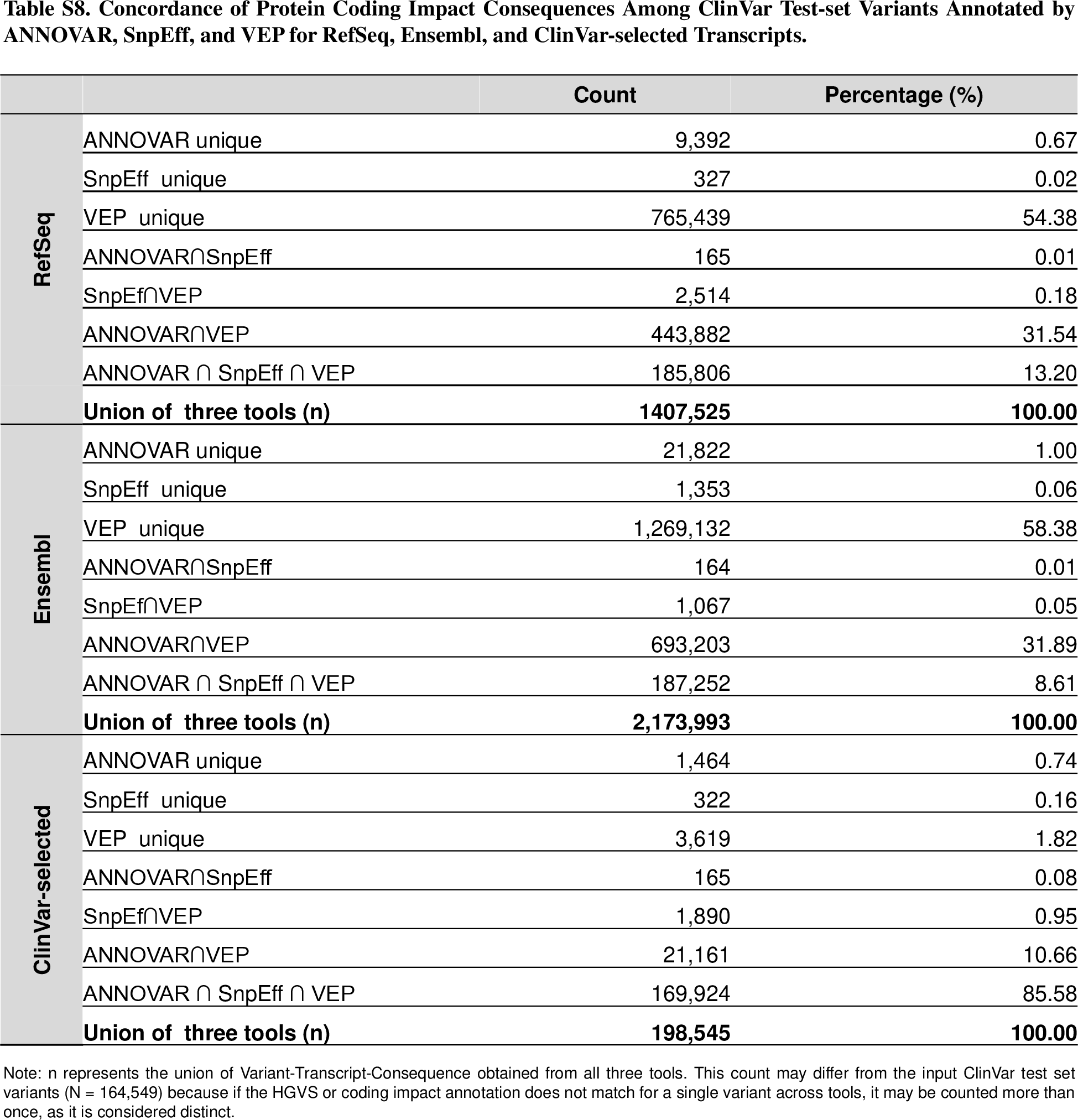

Supplement: Supplementary file 8 — Additional file 8 [file 40246_2025_778_MOESM8_ESM.tif]

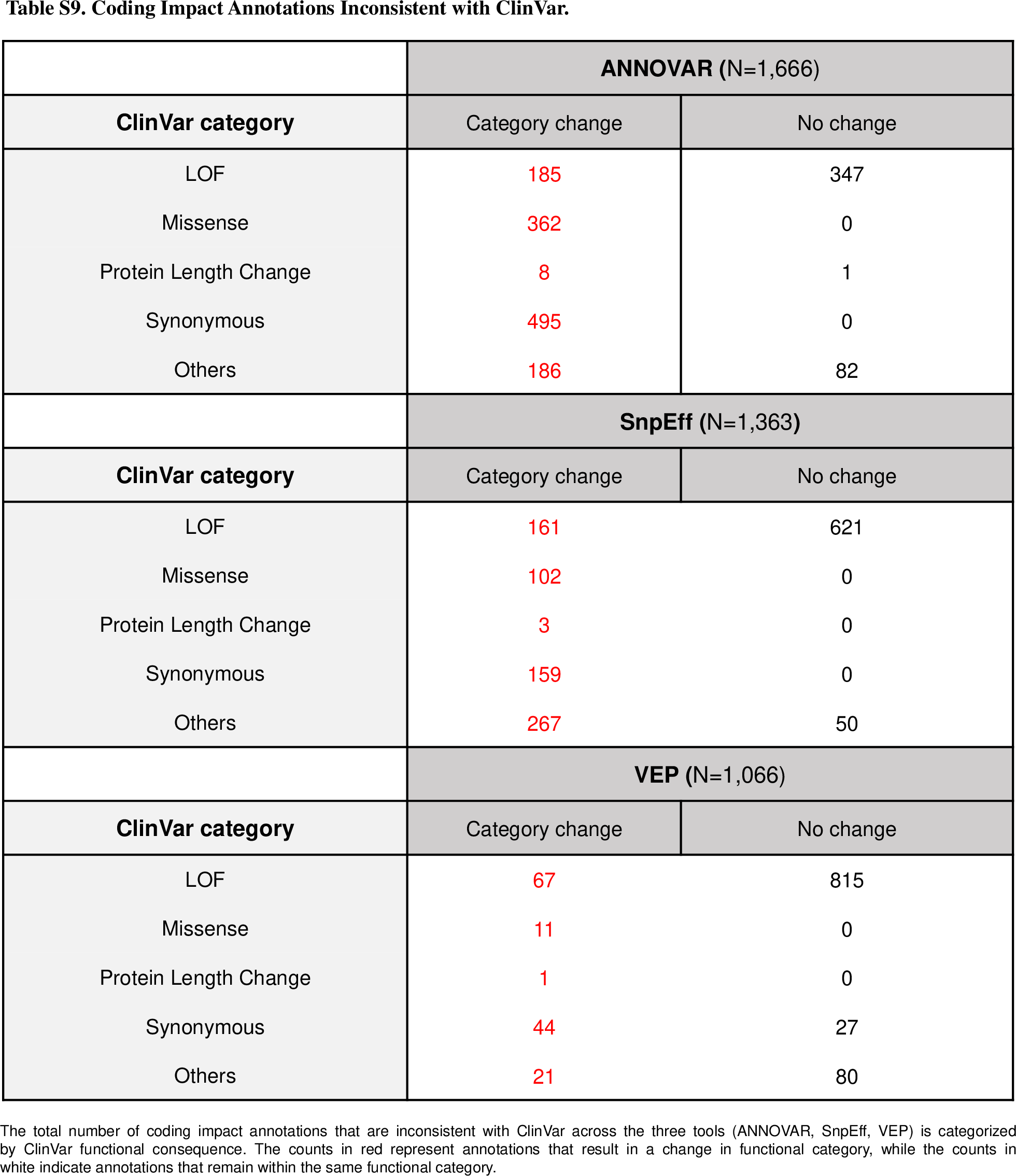

Supplement: Supplementary file 9 — Additional file 9 [file 40246_2025_778_MOESM9_ESM.tif]

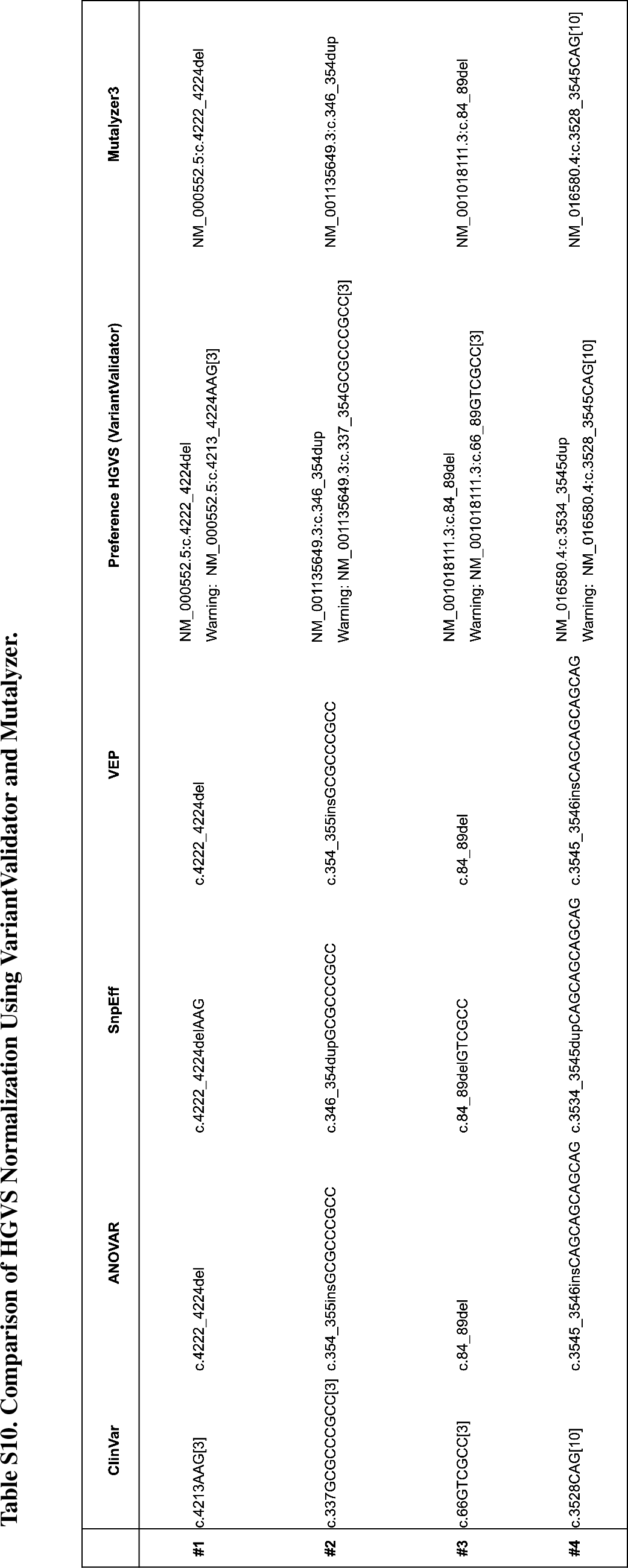

Supplement: Supplementary file 10 — Additional file 10 [file 40246_2025_778_MOESM10_ESM.tif]

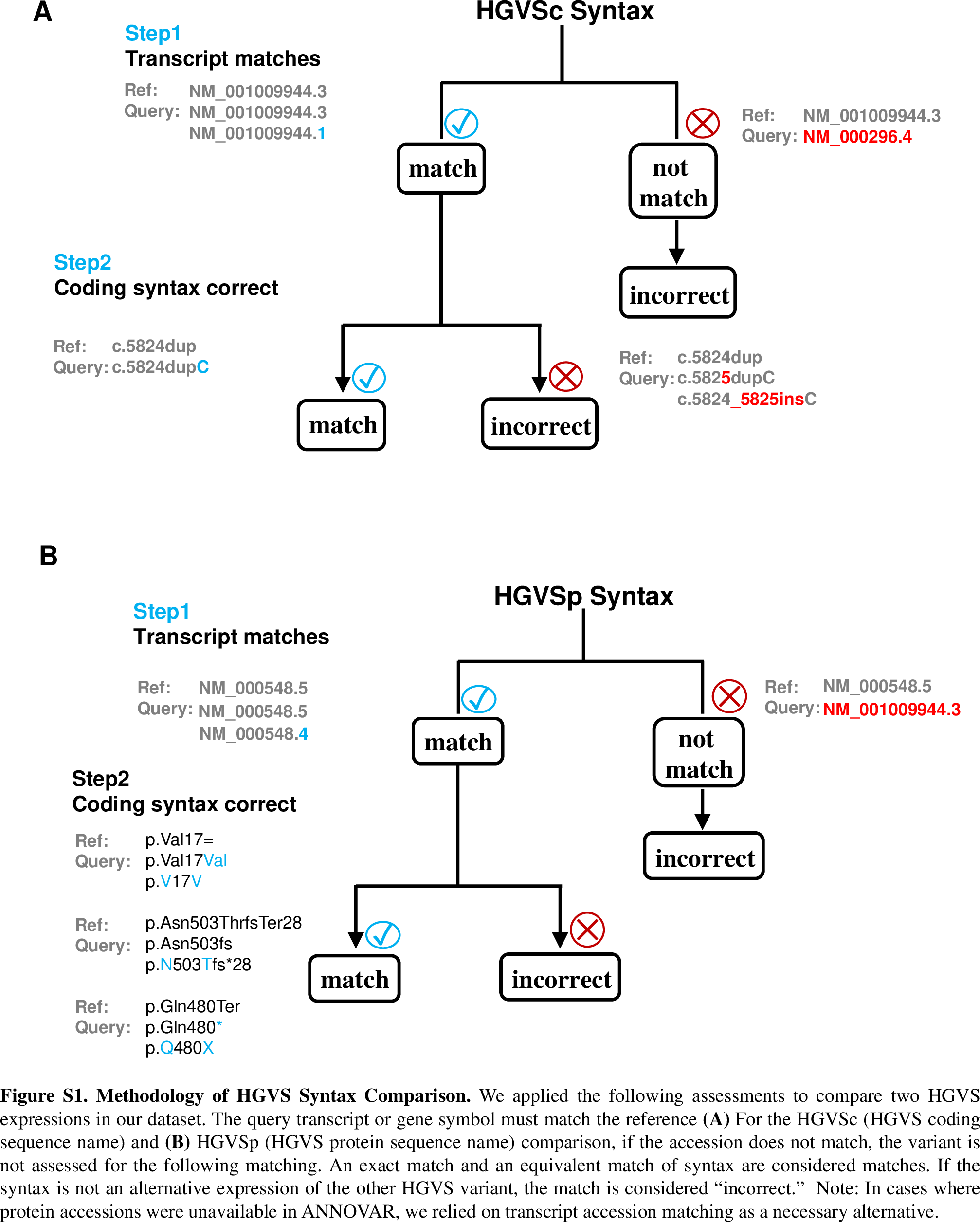

Supplement: Supplementary file 11 — Additional file 11 [file 40246_2025_778_MOESM11_ESM.tif]

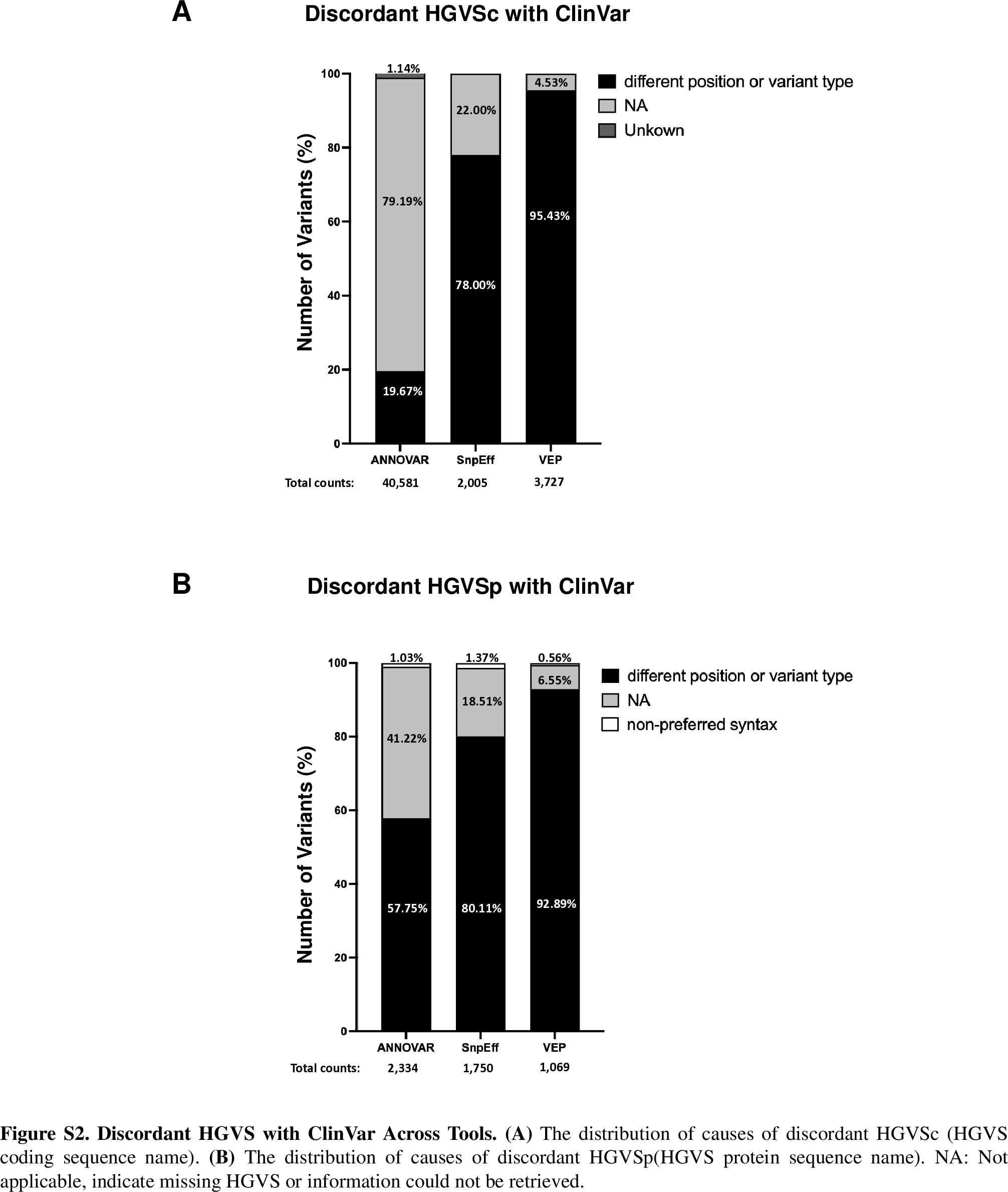

Supplement: Supplementary file 12 — Additional file 12 [file 40246_2025_778_MOESM12_ESM.tif]

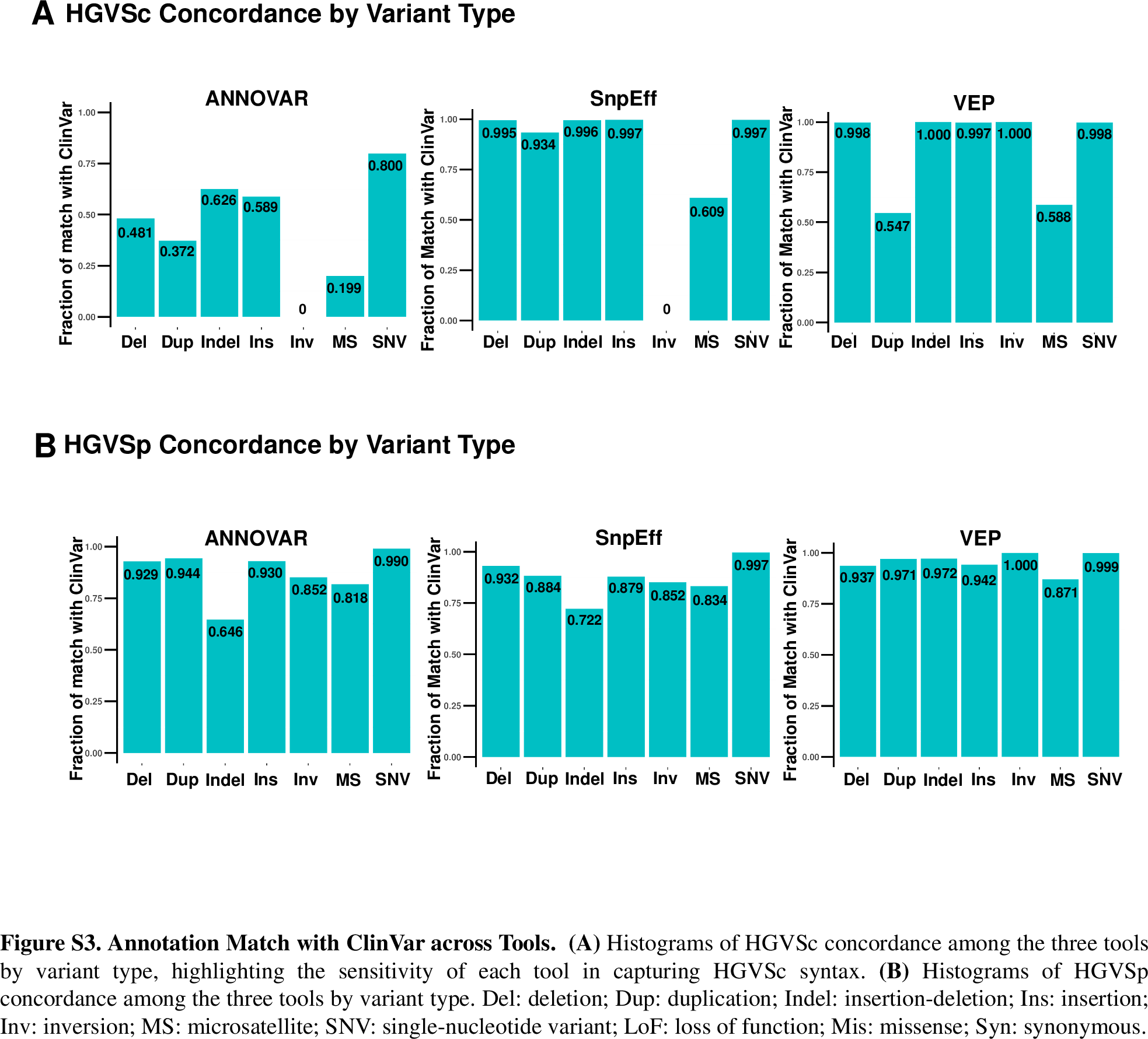

Supplement: Supplementary file 13 — Additional file 13 [file 40246_2025_778_MOESM13_ESM.tif]

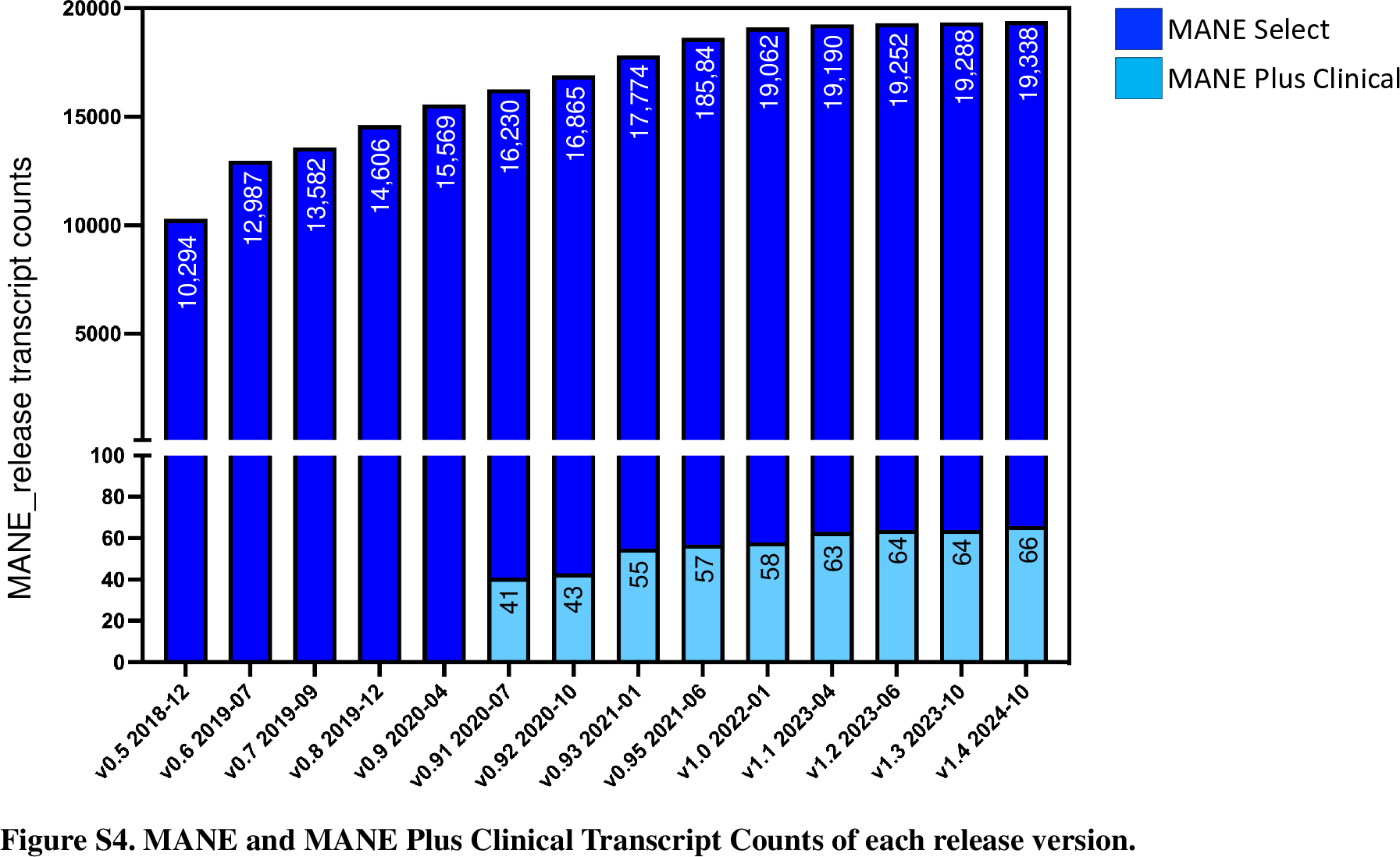

Supplement: Supplementary file 14 — Additional file 14 [file 40246_2025_778_MOESM14_ESM.tif]

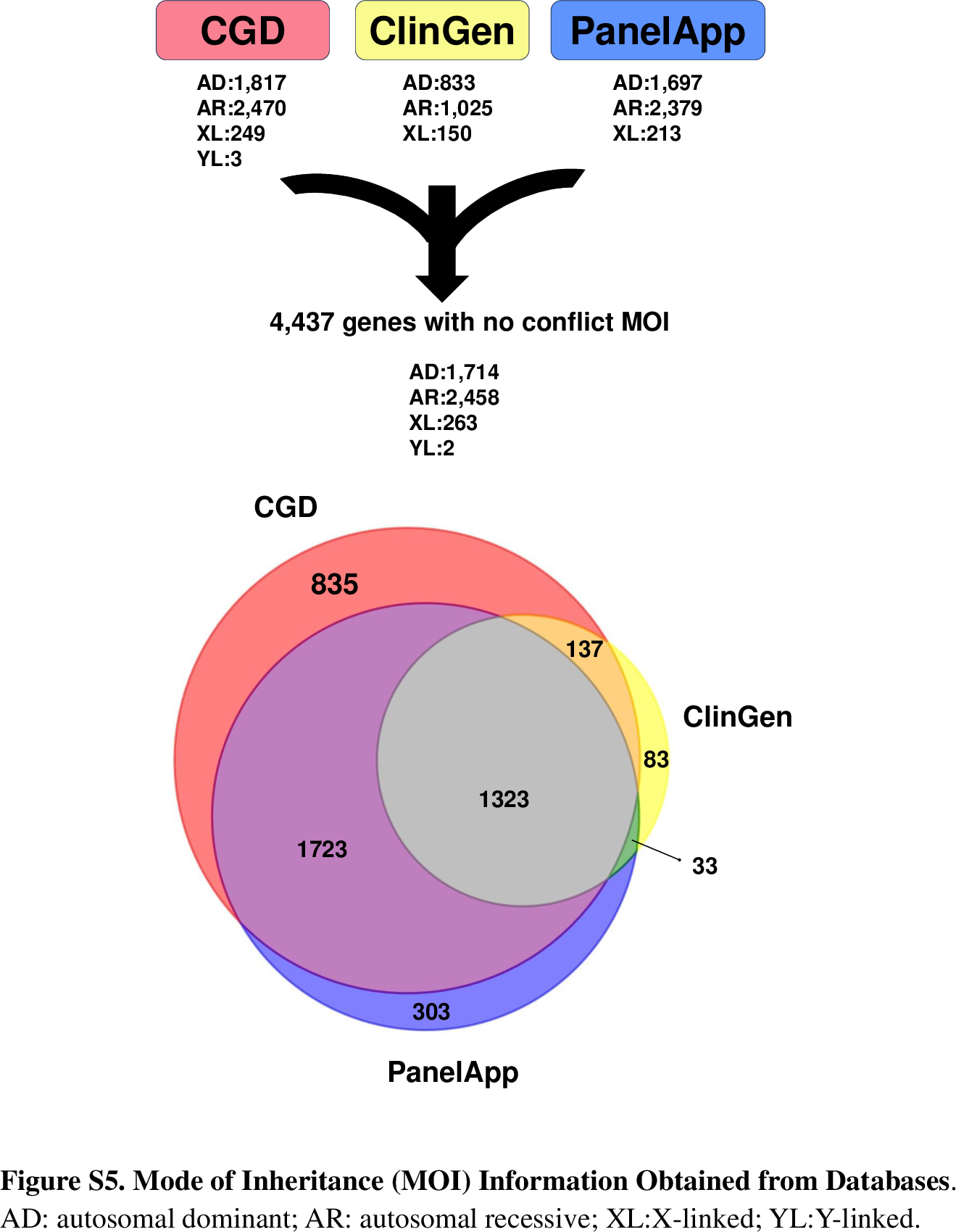

Supplement: Supplementary file 15 — Additional file 15 [file 40246_2025_778_MOESM15_ESM.tif]
