# Supplementary material for: Toward streamline variant classification: discrepancies in variant nomenclature and syntax for ClinVar pathogenic variants across annotation tools
Source: Hum Genomics. 2025 Jun 21;19:70. doi: 10.1186/s40246-025-00778-x (PMC12181866; doi:10.1186/s40246-025-00778-x)
Supplement: Supplementary file 22 — Additional file 22 [file 40246_2025_778_MOESM22_ESM.pdf]

ClinVar

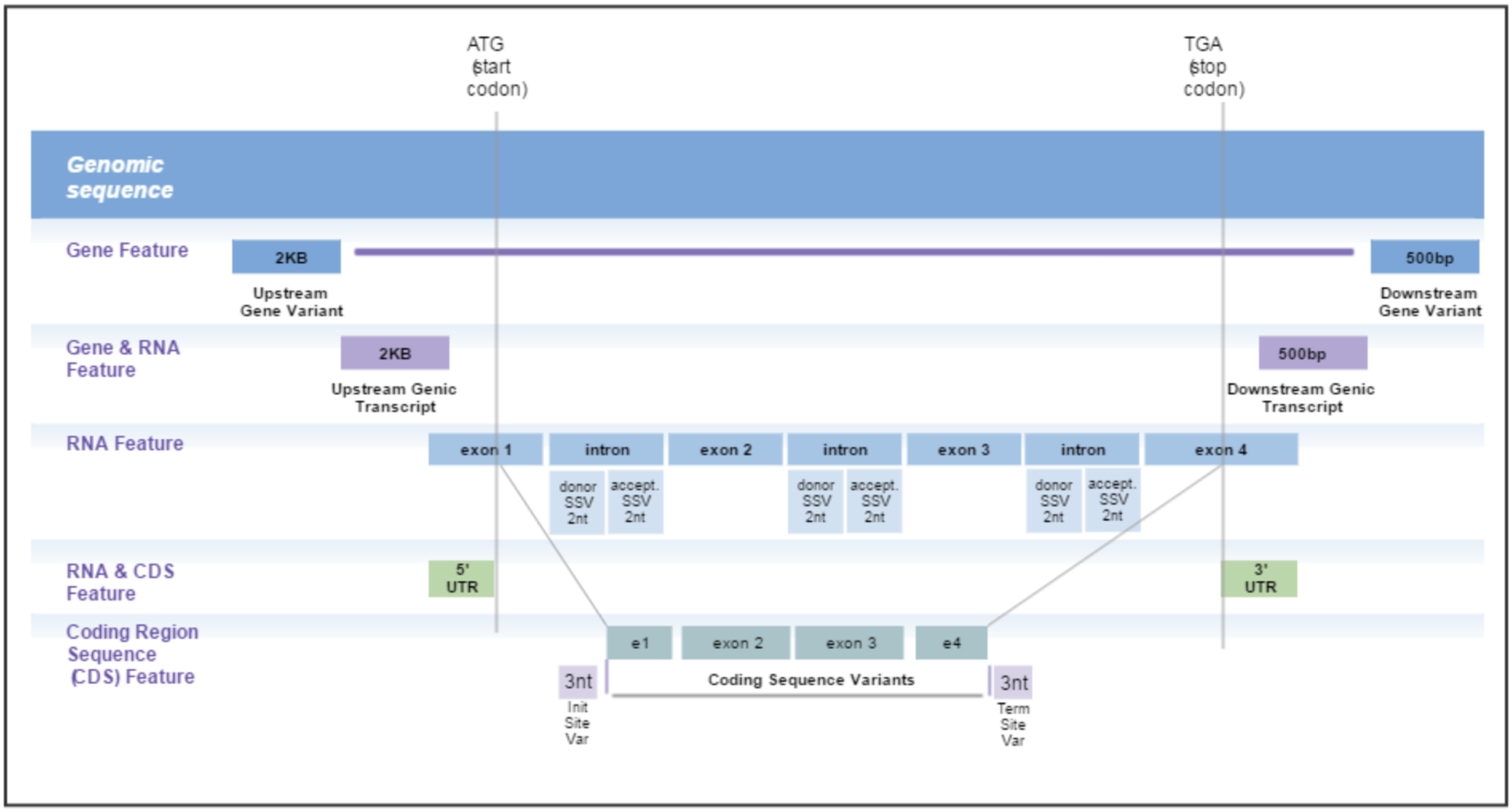

Fig 1. Gene, RNA and Coding Region Sequence (CDS) feature locations used for assigning SO terms

Molecular consequence

Molecular consequence represents effects on protein products from the alterations in the coding nucleotide sequence. NCBI computes molecular consequence, and also assigns location-based ontology terms established by Sequence Ontology (SO), based on where the variant is located relative to gene, RNA and/or coding regions.

Effect on protein products per transcript

For each RNA for which the variant coincides in part or completely within a coding region, we would assign one of the following molecular consequences, as a computed effect of a sequence change on a particular protein product.

Public Term	SO id and value	VCF Tag
Stop Lost	SO:0001578:stop_lost	
Nonsense	SO:0001587:stop_gained	NSN
Synonymous	SO:0001819:synonymous_variant	SYN
Missense	SO:0001583:missense_variant	NSM
Frameshift	SO:0001589 :frameshift_variant	NSF
Inframe Insertion	SO:0001821:inframe_insertion	
Inframe Deletion	SO:0001822:inframe_deletion	
Inframe Indel	SO:0001820:inframe_indel	

Location-based Ontology Terms

Location-based Ontology Terms are assigned to a variant whenever any part of its deletion interval (per the representation of variants that considers them to be pairs of deletion and insertion intervals on a sequence) overlaps one of the Gene, RNA Feature or Coding regions (see illustration). If the variant overlaps more than one region or, if multiple transcripts are involved (as would be the case when the region is relative to a genomic location), all relevant SO terms are reported, in no particular order.

Public Term	SO id and value	VCF Tag
2KB Upstream	SO:0001636 :2KB_upstream_variant	R5
500 bp Downstream	SO:0001634:500B_downstream_variant	R3
3' UTR	SO:0001624 :3_prime_UTR_variant	U3
5' UTR	SO:0001623 :5_prime_UTR_variant	U5
Coding Sequence Variant	SO:0001580 :coding_sequence_variant	
Initiator Codon	SO:0001582 :initiator_codon_variant	
Terminator Codon	SO:0001590:terminator_codon_variant	
500 bp Downstream Genic Variant	SO:0002152:genic_downstream_transcript_variant	
2KB Upstream Genic Variant	SO:0002153:genic_upstream_transcript_variant	
Intron	SO:0001627:intron_variant	INT
Non Coding Transcript Variant	SO:0001619 :non_coding_transcript_variant	
Splice Acceptor	SO:0001574 :splice_acceptor_variant	ASS
Splice Donor	SO:0001575 : splice_donor_variant	DSS

The information was obtained from ClinVar: https://www.ncbi.nlm.nih.gov/variation/docs/glossary/

ANNOVAR

Value	Default precedence	Explanation	Sequence Ontology
exonic	1	variant overlaps a coding	exon_variant (SO:0001791)
splicing	1	variant is within 2-bp of a splicing junction (use -splicing_threshold to change this)	splicing_variant (SO:0001568)
ncRNA	2	variant overlaps a transcript without coding annotation in the gene definition (see Notes below for more explanation)	non_coding_transcript_variant (SO:0001619)
UTR5	3	variant overlaps a 5' untranslated region	5_prime_UTR_variant (SO:0001623)
UTR3	3	variant overlaps a 3' untranslated region	3_prime_UTR_variant (SO:0001624)
intronic	4	variant overlaps an intron	intron_variant (SO:0001627)
upstream	5	variant overlaps 1-kb region upstream of transcription start site	upstream_gene_variant (SO:0001631)
downstream	5	variant overlaps 1-kb region downtream of transcription end site (use -neargene to change this)	downstream_gene_variant (SO:0001632)
intergenic	6	variant is in intergenic region	intergenic_variant (SO:0001628)

The value of the first column takes the following precedence (as of December 2010 and later version of ANNOVAR): exonic = splicing > ncRNA> > UTR5/UTR3 > intron > upstream/downstream > intergenic. The precedence defined above is used to decide what function to print out when a variant fit multiple functional categories. Note that:

1. the "exonic" here refers only to coding exonic portion , but not UTR portion, as there are two keywords (UTR5, UTR3) that are specifically reserved for UTR annotations.
2. "splicing" in ANNOVAR is defined as variant that is within 2-bp away from an exon/intron boundary by default, but the threshold can be changed by the --splicing_threshold argument. Before Feb 2013, if "exonic,splicing" is shown, it means that this is a variant within exon but close to exon/intron boundary; this behavior is due to historical reason, when a user requested that exonic variants near splicing sites be annotated with splicing as well. However, I continue to get user emails complaining about this behavior despite my best efforts to put explanation in the ANNOVAR website with details. Therefore, starting from Feb 2013 , "splicing" only refers to the 2bp in the intron that is close to an exon, and if you want to have the same behavior as before, add -exonicsplicing argument.
3. If a variant is located in both 5' UTR and 3' UTR region (possibly for two different genes), then the "UTR5,UTR3" will be p rinted as the output.
4. The term "upstream" and "downstream" is defined as 1-kb away from transcription start site or transcription end site, respectively, taking in account of the strand of the mRNA; the --neargene threshold can be used to adjust this threshold.
5. If a variant is located in both downstream and upstream region (possibly for 2 different genes), then the "upstream,downstream" will be printed as the output. In 2011 June version of ANNOVAR, the splicing annotation is improved. If the splicing site is in intron, then all isoforms and the corresponding base change will be printed.

The information was obtained from ANNOVAR: https://annovar.openbioinformatics.org/en/latest/user-guide/gene/?#switching-to-ucscensembl-gene-annotation

SnpEff

Table 2. Detailed effect list from SnpEff

Effect	Note
INTERGENIC	The variant is in an intergenic region
UPSTREAM	Upstream of a gene (default length: 5K bases)
UTR_5_PRIME	Variant hits 5'UTR region
UTR_5_DELETED	The variant deletes and exon which is in the 5'UTR of the transcript
START_GAINED	A variant in 5'UTR region produces a three base sequence that can be a START codon
SPLICE_SITE_ACCEPTOR	The variant hits a splice acceptor site (defined as two bases before exon start, except for the first exon)
SPLICE_SITE_DONOR	The variant hits a Splice donor site (defined as two bases after coding exon end, except for the last exon)
START_LOST	Variant causes start codon to be mutated into a non-start codon
SYNONYMOUS_START	Variant causes start codon to be mutated into another start codon
CDS	The variant hits a CDS
GENE	The variant hits a gene
TRANSCRIPT	The variant hits a transcript
EXON	The vairant hist an exon
EXON_DELETED	A deletion removes the whole exon
NON_SYNONYMOUS_CODING	Variant causes a codon that produces a different amino acid
SYNONYMOUS_CODING	Variant causes a codon that produces the same amino acid
FRAME_SHIFT	Insertion or deletion causes a frame shift
CODON_CHANGE	One or many codons are changed
CODON_INSERTION	One or many codons are inserted
CODON_CHANGE_PLUS_CODON_INSERTION	One codon is changed and one or many codons are inserted
CODON_DELETION	One or many codons are deleted
CODON_CHANGE_PLUS_CODON_DELETION	One codon is changed and one or more codons are deleted
STOP_GAINED	Variant causes a STOP codon
SYNONYMOUS_STOP	Variant causes stop codon to be mutated into another stop codon
STOP_LOST	Variant causes stop codon to be mutated into a non-stop codon
INTRON	Variant hist and intron. Technically, hits no exon in the transcript
UTR_3_PRIME	Variant hits 3'UTR region
UTR_3_DELETED	The variant deletes and exon which is in the 3'UTR of the transcript
DOWNSTREAM	Downstream of a gene (default length: 5K bases)
INTRON_CONSERVED	The variant is in a highly conserved intronic region
INTERGENIC_CONSERVED	The variant is in a highly conserved intergenic region

The information was obtained from Cingolani Pet al. A program for annotating and predicting the effects of single nucleotide polymorphisms, SnpEff: SNPs in the genome of Drosophila melanogaster strain w1118; iso-2; iso-3. Fly (Austin). 2012. 6(2):80-92. doi: 10.4161/fly.19695.

VEP

See below a diagram showing the location of each display term relative to the transcript structure:

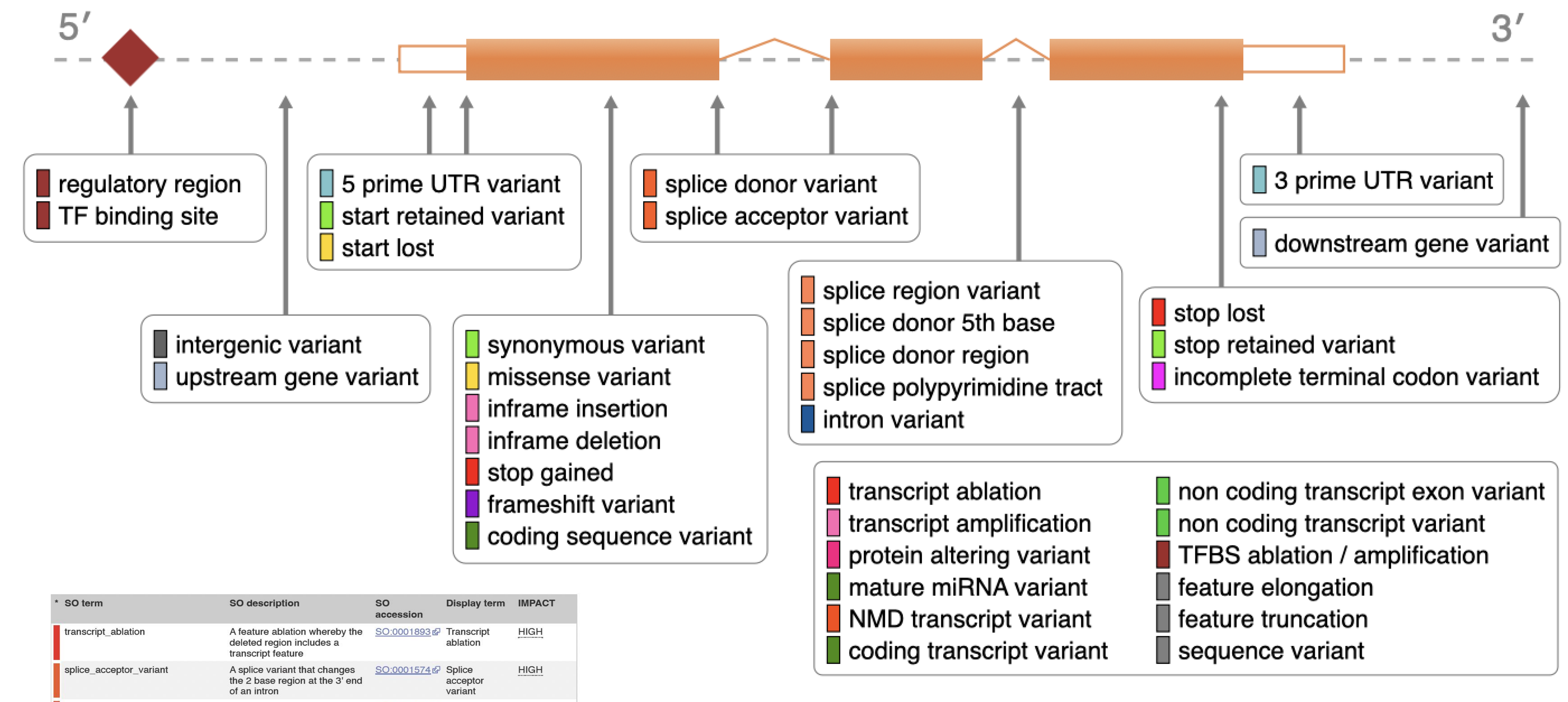

* SO term	SO description	SO accession	Display term	IMPACT
transcript_ablation	A feature ablation whereby the deleted region includes a transcript feature	SO:0001893	Transcript ablation	HIGH
splice_acceptor_variant	A splice variant that changes the 2 base region at the 3' end of an intron	SO:0001574	Splice acceptor variant	HIGH
splice_donor_variant	A splice variant that changes the 2 base region at the 5' end of an intron	SO:0001575	Splice donor variant	HIGH
stop_gained	A sequence variant whereby at least one base of a codon is changed, resulting in a premature stop codon, leading to a shortened transcript	SO:0001587	Stop gained	HIGH
frameshift_variant	A sequence variant which causes a disruption of the translational reading frame, because the number of nucleotides inserted or deleted is not a multiple of three	SO:0001589	Frameshift variant	HIGH
stop_lost	A sequence variant where at least one base of the terminator codon (stop) is changed, resulting in an elongated transcript	SO:0001578	Stop lost	HIGH
start_lost	A codon variant that changes at [illegible] start codon	SO:0002012	Start lost	HIGH
transcript_amplification	A feature amplification of a region containing a transcript	SO:0001889	Transcript amplification	HIGH
feature_elongation	A sequence variant that causes the extension of a genomic feature, with regard to the reference sequence	SO:0001907	Feature elongation	HIGH
feature_truncation	A sequence variant that causes the reduction of a genomic feature, with regard to the reference sequence	SO:0001906	Feature truncation	HIGH
inframe_insertion	An inframe non synonymous variant that inserts bases into in the coding sequence	SO:0001821	Inframe insertion	MODERATE
inframe_deletion	An inframe non synonymous variant that deletes bases from the coding sequence	SO:0001822	Inframe deletion	MODERATE
missense_variant	A sequence variant, that changes one or more bases, resulting in a different amino acid sequence but where the length is preserved	SO:0001583	Missense variant	MODERATE
protein_altering_variant	A sequence_variant which is predicted to change the protein encoded in the coding sequence	SO:0001818	Protein altering variant	MODERATE
splice_donor_5th_base_variant	A sequence variant that causes a change at the 5th base pair after the start of the intron in the orientation of the transcript	SO:0001787	Splice donor 5th base variant	LOW
splice_region_variant	A sequence variant in which a change has occurred within the region of the splice site, either within 1-3 bases of the exon or 3-8 bases of the intron	SO:0001630	Splice region variant	LOW
splice_donor_region_variant	A sequence variant that falls in the region between the 3rd and 6th base after splice junction (5' end of intron)	SO:0002170	Splice donor region variant	LOW
splice_polypyrimidine_tract_variant	A sequence variant that falls in the polypyrimidine tract at 3' end of intron between 17 and 3 bases from the end (acceptor -3 to acceptor -17)	SO:0002169	Splice polypyrimidine tract variant	LOW
incomplete_terminal_codon_variant	A sequence variant where at least one base of the final codon of an incompletely annotated transcript is changed	SO:0001626	Incomplete terminal codon variant	LOW
start_retained_variant	A sequence variant where at least one base in the start codon is changed, but the start remains	SO:0002019	Start retained variant	LOW
stop_retained_variant	A sequence variant where at least one base in the terminator codon is changed, but the terminator remains	SO:0001567	Stop retained variant	LOW
synonymous_variant	A sequence variant where there is no resulting change to the encoded amino acid	SO:0001819	Synonymous variant	LOW
coding_sequence_variant	A sequence variant that changes the coding sequence	SO:0001580	Coding sequence variant	MODIFIER
mature_miRNA_variant	A transcript variant located with the sequence of the mature miRNA	SO:0001620	Mature miRNA variant	MODIFIER
5_prime_UTR_variant	A UTR variant of the 5' UTR	SO:0001623	5 prime UTR variant	MODIFIER
3_prime_UTR_variant	A UTR variant of the 3' UTR	SO:0001624	3 prime UTR variant	MODIFIER
non_coding_transcript_exon_variant	A sequence variant that changes non-coding exon sequence in a non-coding transcript	SO:0001792	Non coding transcript exon variant	MODIFIER
intron_variant	A transcript variant occurring within an intron	SO:0001627	Intron variant	MODIFIER
NMD_transcript_variant	A variant in a transcript that is the target of NMD	SO:0001621	NMD transcript variant	MODIFIER
non_coding_transcript_variant	A transcript variant of a non coding RNA gene	SO:0001619	Non coding transcript variant	MODIFIER
coding_transcript_variant	A transcript variant of a protein coding gene	SO:0001968	Coding transcript variant	MODIFIER
upstream_gene_variant	A sequence variant located 5' of a gene	SO:0001631	Upstream gene variant	MODIFIER
downstream_gene_variant	A sequence variant located 3' of a gene	SO:0001632	Downstream gene variant	MODIFIER
TFBS_ablation	A feature ablation whereby the deleted region includes a transcription factor binding site	SO:0001895	TFBS ablation	MODIFIER
TFBS_amplification	A feature amplification of a region containing a transcription factor binding site	SO:0001892	TFBS amplification	MODIFIER
TF_binding_site_variant	A sequence variant located within a transcription factor binding site	SO:0001782	TF binding site variant	MODIFIER
regulatory_region_ablation	A feature ablation whereby the deleted region includes a regulatory region	SO:0001894	Regulatory region ablation	MODIFIER
regulatory_region_amplification	A feature amplification of a region containing a regulatory region	SO:0001891	Regulatory region amplification	MODIFIER
regulatory_region_variant	A sequence variant located within a regulatory region	SO:0001566	Regulatory region variant	MODIFIER
intergenic_variant	A sequence variant located in the intergenic region, between genes	SO:0001628	Intergenic variant	MODIFIER
sequence_variant	A sequence_variant is a non exact copy of a sequence_feature or genome exhibiting one or more sequence_alteration	SO:0001060	Sequence variant	MODIFIER

* Corresponding colours for the Ensembl web displays.

Missense variants may have further annotation on their effect on the protein function, using a number of algorithms.

The information was obtained from Ensembl: https://asia.ensembl.org/info/genome/variation/prediction/predicted_data.html
